# MOVING, THINKING, AND SLEEPING: NOVEL INSIGHTS INTO PHYSICAL AND COGNITIVE HEALTH FROM ACCELEROMETRY DATA

**DOI:** 10.1093/geroni/igac059.1303

**Published:** 2022-12-20

**Authors:** Jennifer Schrack, Amal Wanigatunga

## Abstract

Physical activity and sleep are well-established predictors of health and longevity with aging. Wrist accelerometers, that produce high-frequency time series data, capture multiple aspects of daily physical activity and sleep 24-hours/day. Historically, the majority of accelerometry-based activity research has employed summary metrics to understand the associations of total daily physical activity and sleep with physical and cognitive health. Although these measures are important for understanding conformity with physical activity and sleep recommendations, they underutilize the potential of these data. Further, the summary metrics may differ by accelerometer type/brand, making it difficult to translate results across device types and studies. This symposium will examine the associations between accelerometry-derived physical activity and various aging-related health outcomes, and compare the measurement properties of two commonly used accelerometers for measuring sleep. Ms. Marino will discuss the association of physical activity volume and fragmentation with the presence of the Apolipoprotein-ε4 genotype in the Baltimore Longitudinal Study of Aging (BLSA), overall and by time of day. Dr. Wanigatunga will present evidence on the association of physical activity patterns with beta amyloid plaques in the BLSA. Dr. Schrack will present the association of physical activity fragmentation and diurnal patterns with peripheral artery disease in the Hispanic Community Health Study/Study of Latinos (HCHS/SOL). Finally, Ms. Liu will compare measurement of sleep variables derived from two commonly used accelerometers. Collectively, these presentations highlight ways to utilize the richness of accelerometry data to illuminate more sensitive associations between movement and health outcomes to advance prevention science and promote health aging.

